# Perceived knowledge, attitudes and practices regarding the medical consortium among medical staff in Sichuan, China: a cross-sectional survey

**DOI:** 10.1186/s12913-023-10146-x

**Published:** 2023-11-29

**Authors:** Wenqi Zeng, Wenjuan Tao, Yanlin Yang, Yong Li, Bingqing Lu, Qian Zhao, Zhuyue Li, Miao Wang, Zhanglin Shui, Jin Wen

**Affiliations:** 1 Institute of Hospital Management, West China Hospital, Sichuan University, Chengdu, Sichuan China; 2 Evidence-based Medical Center, Hospital of Chengdu University of Traditional Chinese Medicine (Traditional Chinese Medicine Hospital of Sichuan), Chengdu, Sichuan China; 3 Department of Medical Administration, Chengdu Municipal Health Commission, Chengdu, Sichuan China; 4 West China School of Nursing/West China Hospital, Sichuan University, Chengdu, China; 5 Department of Public Affairs Development, West China Hospital, Sichuan University, Chengdu, China; 6 Outpatient Department, West China Hospital, Sichuan University, Chengdu, Sichuan China

**Keywords:** KAP, Medical Consortium, Medical staff, Integrated Health Care

## Abstract

**Background:**

In China, fragmented and inefficient health care systems are common while quality resources are limited. To promote an organized, efficient system, the government launched a medical consortium policy to vertically integrate health care through the collaboration of different levels of medical care. Logically, medical staff’s knowledge, attitudes and practices (KAP) regarding the consortium are critical for its development. The objective of this study was to explore the KAP regarding the medical consortium among medical staff in a medical consortium in Sichuan Province, China.

**Methods:**

A cross-sectional survey was conducted. In total, 690 medical staff members in 3 cities of Sichuan Province, China, were interviewed from November 2018 to December 2018. The questionnaire consisted of 18 items, including 4 items related to perceived knowledge, 4 items related to attitudes and 2 items related to practices, and was rated on a 5-point Likert scale (one = strongly disagree/do not know, five = strongly agree/know).

**Results:**

The effective response sample was 640 copies of the questionnaire, and most medical staff members (92.50%) knew about the cooperation with other hospitals in the medical consortium. Medical staff scored differently on each item in the questionnaire, with the highest score being the item ‘agreeing with the ward rounds and clinical teaching and training organized by the leading hospital’ (4.54 ± 0.76), and the lowest score being the item ‘frequency in participating in ward rounds and clinical teaching organized by the leading hospital’ (2.83 ± 1.36). In addition, the effect of demographic characteristics on KAP was evaluated by stepwise multiple regression analysis, and a significant positive correlation was found between all the studied variables by Spearman’s correlation (p < 0.05).

**Conclusions:**

This study showed that the attitudes toward and knowledge of the medical consortium significantly contribute to practices, satisfaction with the support work performed by the leading hospital and agreement of improvement after joining the medical consortium. Thus, to improve medical staff’s KAP and satisfaction, publicity and educational programs in medical consortia are necessary, and the leading hospital should attach importance to the informatization construction and demand of different medical staff members.

**Clinical trial registration:**

There are no clinical trials in this study.

**Supplementary Information:**

The online version contains supplementary material available at 10.1186/s12913-023-10146-x.

## Background [1]

In China, the allocation of health care resources has been imbalanced for a long time, e.g., the reasonable allocation of health care resources across different levels of health care facilities is lacking [1]. The main issue with China’s current health care system is that patients seem to seek care first at tertiary hospitals. This has led to the long-standing “tertiary hospitals squeezing the threshold and the deserted house of the grassroots hospital” phenomenon [2]. This causes long wait times, a disorganized referral system and a reduction in patient care, as the hierarchical treatment system (referral system) is not used to its full potential [3]. In 2017, the State Council of the People’s Republic of China issued guidelines on promoting the development of a new health care policy throughout China, referred to as the ‘Medical Consortium’ (also known as ‘Medical Alliance’ or ‘Medical Union’) [4, 5].

A medical consortium creates a form of vertically Integrated health care through the collaboration of different levels of medical care, typically involving one widely recognized tertiary hospital and several secondary hospitals or community health centers that work together to improve patients’ outcomes [6]. The medical consortium is an important component of hierarchical medical systems. Its establishment is an effective exploration and practice model for directing high-quality medical resources to primary care institutions, promoting integrated health care such as medical treatment, rehabilitation and care, achieving homogeneous medical quality and reducing medical costs [7].

The function of integrated health care is described by the World Health Organization as “health services that are managed and delivered in a way that ensures people receive a continuum of health promotion, disease prevention, diagnosis, treatment, disease management, rehabilitation, and palliative care services, at the different levels and sites of care within the health system, and according to their needs throughout their life course” [8]. There are expectations that through fulfilling primary care functions, integrated health care will improve health care, enhance health outcomes and reduce costs [9]. At the same time, the medical consortium conforms to the global trend of integrated health care or integrated delivery systems that build on the functions of primary care, namely, first contact and continuous, coordinated and comprehensive care [10].

To completely promote the development of the medical consortium, a great deal of research has emerged in recent years in China. Much of the early research focused on the policy analysis of the medical consortium [11], exploration and practice of the medical consortium model [7, 12, 13], and the first visit and referral in the medical consortium [14, 15]. However, little evidence exists for how health care providers view the medical consortium from the stakeholder’s perspective.

Judging from the practical experience of the past years, this cooperative model in the medical consortium has achieved a multiwin situation, including among medical staff, patients, hospitals, the health care administrative agency, and the medical insurance bureau [13]. Nevertheless, recent studies showed that the hospitals or medical consortia of some countries and regions do not seem to apparently improve continuity, coordination or quality of care [16–20]; therefore, hospitals or medical consortia need to be developed immediately in preparation for future challenges. Medical staff members are the most important individuals for medical consortium success and are also the first responders to patients. Recent researches have revealed that employees lack proper knowledge, demonstrate negative attitudes, and engage in unacceptable practices in certain facets of their work [21–23]. Consequently, Medical staffs’ knowledge, attitudes and practices (KAP) have a direct impact on regional or national health care delivery.

The KAP model is among the most widely used models in the medical field. The model was first used during the middle of the nineteenth century to assess family planning and population, and the model suggests that any practices (behaviors) are determined by the person’s attitudes and knowledge toward those behaviors [24]. Understanding the KAP of medical staff regarding the medical consortium will help promote the implementation and improvement of the medical consortium and realize an integrated health care system. However, few studies have comprehensively analyzed the KAP regarding medical consortia among medical staff in China. The primary purpose of this study was to investigate the perceived knowledge, attitudes, and practices of medical staff regarding medical consortia.

## Methods

### Setting and participants

West China Hospital of Sichuan University (WCH) located in western China is a prestigious and well-known medical center in Chengdu, Sichuan Province, and has been ranked as the second best hospital in China during the last 10 years. WCH launched the WCH medical consortium and, as a leading hospital, played the core role and interacted with different levels of hospitals in the hospital networks across different regions. Here, we chose medical staff from the WCH medical consortium to participate in this study, including doctors, nurses, medical technicians and administrators.

### Sampling methods

In the hospitals belonging to the WCH medical consortium, there were 20,000 medical staff members. The target was to achieve a 95% confidence interval with a 5% acceptable margin of error and a 1.2 factor for clustering, accordingly, the ideal sample size comprised 453 randomly selected medical staff members. Three hospitals were selected that were the first to join the WCH medical consortium and had been working together for the longest time; therefore, we investigated the medical staff in 3 cities (Ziyang, Guangan, Jintang County in Chengdu) by convenience sampling from November 2018 to December 2018. To prevent sample loss and other factors, 690 individuals were interviewed, after excluding inconsistent, unknown, or other unreliable data, the final sample size was 640 individuals.

### Survey tool

The questionnaire consisted of 18 items, including 4 items related to perceived knowledge, 4 items related to attitudes and 2 items related to practices. In addition, 6 items for demographic data, satisfaction about the leading hospital and perceived improvement of the hospital were surveyed. The respondents were asked to select their level of agreement/knowledge (a five-point Likert scale where one = strongly disagree/do not know to five = strongly agree/know) for each item (except the demographic questions). The retest reliability of the presurvey was 0.813, and the Cronbach’s alpha coefficient was 0.827, suggesting that the reliability of the questionnaire was very high. The questionnaire was recognized by medical consortium experts and investigators, which suggested that the validity of the questionnaire was fair. The questionnaire included items on general conditions, **perceived knowledge** (including knowing about the cooperation with other hospitals in the medical consortium, the concept of the medical consortium, knowing what the leading hospital was doing to support the medical consortium, and whether a hierarchical medical system could be realized through the medical consortium), **attitudes** (including supporting the hospital in joining a medical consortium, agreeing to the ward rounds and clinical teaching and training organized by the leading hospital, agreeing to the informatization construction in the medical consortium, agreeing to mutual recognition of inspection results in the medical consortium), **practices** (including the frequency of participating in ward rounds and clinical teaching organized by the leading hospital and the frequency of participating in activities organized by the leading hospital) and other questions (including satisfaction regarding the support work done by the leading hospital for its members and whether the hospital improved after joining the medical consortium).

### Data collection

Trained, qualified investigators were separately sent to each hospital. After gathering the medical staff in the hospital, the investigators explained the purpose of this survey and the instructions for the questionnaire. Then, the medical staff surveyed completed the anonymous self-administered questionnaires, and all participants provided verbal informed consent. The investigators collected the questionnaires on-site. Ethical principles were observed throughout.

### Statistical analysis

SPSS 23.0 statistical software (Version 23.0; IBM Corp., Armonk, NY, USA) was used for statistical analysis. Categorical variables are presented as percentages. Numerical variables are presented as the means ± standard deviations (means ± SDs). Perceived knowledge, attitudes and practice-associated factors were analyzed using linear stepwise regression analysis. The dependent variable (y) represented the score of perceived knowledge, attitudes and practices. Independent variables were gender (x1, male = 0, female = 1), age (years) (x2), length of service (years) (x3), job type (x4, doctors = 0, nurses = 1, x5, doctors = 0, medical technicians = 1, x6, doctors = 0, administrators = 1), education level (x7), and titles (x8); gender and job type were coded as dummy variables. Correlation was evaluated by using Spearman’s test. P < 0.05 was considered statistically significant.

## Results

A total of 690 survey questionnaires were distributed, and 640 valid questionnaires were collected (response rate: 92.75%), for which 155 of the respondents were male (24.22%) and 485 were female (75.78%). The average age was 33.31 ± 7.85 years (ranging from 20 to 60 years). The median length of service of the medical staff surveyed was 9.76 ± 8.12 years (ranging from 1 to 37 years). There were 193 people (30.16%) who had worked less than 5 years, over half of the people (50.63%) had worked 5–15 years, and 123 people (19.22%) had worked for more than 15 years. The majority of the respondents in this study were nurses (51.41%), followed by doctors (37.97%), and the rest were medical technicians (5.63%) and administrators (5.00%). Most of the respondents had a baccalaureate degree (70.63%), whereas 42 of the respondents had a master’s degree or higher (6.56%). Additionally, the majority of the respondents were junior employees (59.06%), and 94 were senior employees (14.69%) (Table 1).

**Table 1:** Demographic characteristics of the respondents

Items		Total frequency (n)	Percentage (%)
**Gender**	Male	155	24.22
	Female	485	75.78
**Age (years)**	< 30	248	38.75
	30–45	331	51.72
	> 45	61	9.53
**Length of service (years)**	< 5	193	30.16
	5–15	324	50.63
	> 15	123	19.22
**Job type**	Doctors	243	37.97
	Nurses	329	51.41
	Medical technicians	36	5.63
	Administrators	32	5.00
**Education**	College	146	22.81
	Baccalaureate	452	70.63
	Master’s or higher	42	6.56
**Title**	Junior	378	59.06
	Intermediate	168	26.25
	Senior	94	14.69

### Perceived knowledge about the medical consortium

Regarding the grasp of perceived knowledge (Table 2), most medical staff (92.50%) knew about the cooperation with other hospitals in the medical consortium, whereas the score for all medical staff regarding grasping the concept of the medical consortium was the lowest (3.40 ± 0.99). The highest score of perceived knowledge was for the item ‘a hierarchical medical system can be realized through the medical consortium’ (4.20 ± 1.18). Stepwise multiple regression analysis of perceived knowledge-associated factors was performed and showed that the factors related to perceived knowledge were length of service (years), gender, and education level (p < 0.05), and the regression equation was written as y = 10.774 + 0.057 * x3–0.627 * x1 + 0.451 * x7, as shown in Table 3.

**Table 2:** Perceived knowledge, attitudes and practices of medical staff regarding the medical consortium

Questionnaire statement	Results
**Perceived knowledge**	
Knowing about the cooperation with other hospitals in the medical consortium (%)	92.50
Concept of the medical consortium (Mean ± SD)	3.40 ± 0.99
Knowing what the leading hospital is doing to support the medical consortium (Mean ± SD)	3.49 ± 0.96
A hierarchical medical system can be realized through the medical consortium (Mean ± SD)	4.20 ± 1.18
**Attitudes**	
Supporting the hospital to join a medical consortium (Mean ± SD)	4.21 ± 0.79
Agreeing to the ward rounds and clinical teaching and training organized by the leading hospital (Mean ± SD)	4.54 ± 0.76
Agreeing to the informatization construction in the medical consortium (Mean ± SD)	3.59 ± 0.96
Agreeing to mutual recognition of inspection results in the medical consortium (Mean ± SD)	3.71 ± 0.94
**Practices**	
Frequency of participation in ward rounds and clinical teaching organized by the leading hospital (Mean ± SD)	2.83 ± 1.36
Frequency of participation in activities organized by the leading hospital (Mean ± SD)	3.44 ± 1.27

**Table 3:** Stepwise multiple regression analysis

	Items	β	S.E.	Beta	t value	P value
**Perceived knowledge**	Constant	10.774	0.392		27.517	0.000
	Length of service (years)	0.057	0.012	0.187	4.765	0.000
	Gender	-0.627	0.231	-0.108	-2.710	0.007
	Education level	0.451	0.193	0.094	2.334	0.020
**Attitudes**	Constant	15.631	0.145		108.165	0.000
	Nurse	0.700	0.196	0.144	3.566	0.000
	Administrator	0.994	0.451	0.089	2.207	0.028
**Practices**	Constant	5.834	0.143		40.815	0.000
	Length of service (years)	0.027	0.009	0.114	2.912	0.004
	Nurse	0.336	0.152	0.087	2.207	0.028

### Attitudes regarding the medical consortium

The attitude evaluation (Table 2) showed that the highest score based on the self-assessment questionnaires was for the item ‘agreeing with the ward rounds and clinical teaching and training organized by the leading hospital’ (4.54 ± 0.76), and the lowest score was for the item ‘agreeing with the informatization construction in the medical consortium’ (3.59 ± 0.96). Stepwise multiple regression analysis of attitude-associated factors was performed. The results showed that the factor related to attitudes was job type (p < 0.05), and the regression equation was written as y = 15.631 + 0.700 * x4 + 0.994 * x6, as shown in Table 3.

### Practices regarding the medical consortium

Regarding practices (Table 2), the item ‘frequency of participation in ward rounds and clinical teaching organized by the leading hospital’ had the lowest score (2.83 ± 1.36) in the questionnaire. The score for the item ‘frequency of participation in activities organized by the leading hospital’ was 3.44 ± 1.27. Stepwise multiple regression analysis of practice-associated factors was performed and showed that the factors related to practices were length of service (years) and job type (p < 0.05), and the regression equation was written as y = 5.834 + 0.027 * x3 + 0.336 * x4, as shown in Table 3.

### Spearman’s correlation based on the results

Some questions were investigated to evaluate the current status of the medical consortium. For instance, ‘satisfaction about the support work done by the leading hospital for its members’ scored 3.85 ± 0.85, and ‘the hospital improved after joining the medical consortium’ scored 3.74 ± 0.90. When evaluating all the studied variables, a significant positive correlation was found between perceived knowledge, attitudes, practices and other questions (p < 0.001) (Table 4).

**Table 4:** Spearman’s correlation between perceived knowledge, attitudes, practices and other questions regarding the medical consortium

		Knowledge	Attitudes	Practices	Satisfaction^a^	Perceived improvement^b^
**Knowledge**	**Correlation coefficient**		0.464	0.356	0.454	0.447
	**Sig. (2-tailed)**		p < 0.001	p < 0.001	p < 0.001	p < 0.001
	**n**		640	640	640	640
**Attitudes**	**Correlation coefficient**	0.464		0.391	0.574	0.487
	**Sig. (2-tailed)**	p < 0.001		p < 0.001	p < 0.001	p < 0.001
	**n**	640		640	640	640
**Practices**	**Correlation coefficient**	0.356	0.391		0.384	0.302
	**Sig. (2-tailed)**	p < 0.001	p < 0.001		p < 0.001	p < 0.001
	**n**	640	640		640	640
**Satisfaction** ^**a**^	**Correlation coefficient**	0.454	0.574	0.384		0.545
	**Sig. (2-tailed)**	p < 0.001	p < 0.001	p < 0.001		p < 0.001
	**n**	640	640	640		640
**Perceived Improvement** ^**b**^	**Correlation coefficient**	0.447	0.487	0.302	0.545	
	**Sig. (2-tailed)**	p < 0.001	p < 0.001	p < 0.001	p < 0.001	
	**n**	640	640	640	640	

^a^ Satisfaction about the support work done by the leading hospital for its members

^b^ The hospital improved after joining the medical consortium

## Discussion

This study investigated the KAP regarding medical consortia from the perspectives of medical staff that, in this anonymous questionnaire survey of 640 medical staff members of the WCH medical consortium conducted to explore their KAP regarding the medical consortium, the medical staff comprised mostly women and represented a shortage of higher-titled or more educated staff. The study yielded the following major findings. First, the KAP of the medical staff regarding medical consortia were not satisfactory, especially the low score for practices. Second, perceived knowledge was influenced by length of service, gender and education level, and behavior was influenced by length of service and job type, but attitude was influenced only by job type. Third, there was a significant positive correlation between perceived knowledge, attitudes, and practices and satisfaction with the leading hospital’s support in the medical consortium and perceived hospital improvement.

Regarding perceived knowledge, the majority of the respondents knew about the cooperation with other hospitals in the medical consortium, and the score for the item ‘a hierarchical medical system can be realized through the medical consortium’ was high, whereas the score for the item about the concept of the medical consortium was the lowest in the self-assessment of the knowledge of the medical staff. Furthermore, the score for the item ‘knowing what the leading hospital is doing to support the medical consortium’ was low. Stepwise multiple regression analysis showed that the influence of the factors related to perceived knowledge was ranked in descending order as follows: education, length of service, and gender. Specifically, staff with higher education, longer service duration, and males had stronger cognition of the medical consortium. Our finding that some medical staff members may have poor cognition of professional knowledge is consistent with the literature on this topic [25–27], and this issue may be exacerbated as employee structures are changing dramatically [28]. Based on the above, hospitals need to strengthen publicity and implement educational programs in medical consortia, especially for less educated staff, new staff, and female staff.

The attitude evaluation showed that medical staff were satisfied with the ward rounds and clinical teaching and training organized by the leading hospital, and this was the highest score in the questionnaire. Moreover, the medical staff were mainly in favor of supporting the hospital to join a medical consortium. Stepwise multiple regression analysis of attitudes indicated that they were only influenced by job type. Specifically, nurses had more positive attitudes toward the medical consortium compared to doctors, and administrators had more positive attitudes compared to doctors. However, the informatization construction in the medical consortium scored the worst in terms of attitudes. Findings of recent studies indicated that due to the lack of experience and integrated information systems in medical consortia [29], medical staff of the medical consortia of many countries are not trained in providing integrated health care for patients in a medical consortium [18, 19]. Consequently, the development of more integrated health and care electronic systems is seen as essential not only for the provision of better care to patients but also for the better operation of the medical consortium. An essential enabler will be the free passage of clinical and administrative information between medical staff, patients, and organizations [17, 30].

In practice, the item ‘frequency of participation in ward rounds and clinical teaching organized by the leading hospital’ had the lowest score in the questionnaire. The other item, ‘frequency of participation in activities organized by the leading hospital’, ranked third from the bottom in the questionnaire. In addition, stepwise multiple regression analysis of practices indicated that practices were influenced by length of service and job type. Specifically, staff with longer service duration had stronger practices regarding the medical consortium. Furthermore, nurses had stronger practices compared to doctors. Previous studies illustrated that practices are not as successful as anticipated when policies and measures are not established or emphasized [25, 31]. Medical staff need to be more responsible for treating or servicing patients in hospitals; therefore, hospitals or the medical consortium should pay close attention to the growth of medical staff and formulate ward round and clinical teaching policies to increase their participation and enhance their clinical experience.

To investigate the relationship between different variables, this study demonstrated that as knowledge of the medical consortium increased, medical staff developed a positive attitude, performed better practices, were more satisfied with the support work done by the leading hospital and agreed more that the hospital was improving. This correlation was statistically significant, confirming the previous research that any practices (behaviors) are determined by the person’s attitudes and knowledge toward the behaviors [24]. Moreover, attitude and knowledge may contribute to improving staff’s ability or performance [32–34]. Accordingly, strengthening medical staff’s perceived knowledge and attitudes toward the medical consortium will help strengthen participation behavior and promote the implementation and improvement of the medical consortium; this also necessitates stricter requirements for hospital administrators within the medical consortium to improve the situation. As stepwise multiple regression analysis demonstrated, demographic characteristics, including gender, length of service, job type, and education, influence work-related knowledge, attitudes, and practices, consistent with findings from previous research [28, 35, 36]. Therefore, based on survey results, medical consortia could tailor education frequency and intensity for different groups to improve the homogeneity of internal medical services.

The Global Burden of Disease Study demonstrated that regional performance in health care access and quality was positively associated with economic status [37]. Large disparities in personal health care access and quality have consequently emerged between eastern and western China, partly attributable to the relatively deprived economy in the west [38]. One strength of this study is that it is among the first to comprehensively analyze the KAP regarding medical consortia among medical staff in China. Thus, this study provides foundational insights to inform the development of medical consortia and improvement of access to quality health care nationally, especially in western China. An additional strength is that this is the first study to explore correlations between perceived knowledge, attitudes, and practices about medical consortia. The KAP framework applied here provides a novel methodology that future studies could readily adopt to assess medical professionals within a consortium model.

Our study had several possible limitations: this study focused only on Sichuan Province, China, and the results can only apply in China; and the results from the questionnaire revealed a small effect of demographic characteristics on attitudes and practices, which needs further verification. Due to the gender differences observed in this study, we will aim to control the gender ratio in future studies. Furthermore, we will adopt the mediating effect model to explore whether the main elements of the KAP model (knowledge and attitudes) can significantly predict the variation in the practices of medical staff regarding the medical consortium.

## Conclusions

The findings of this study provide theoretical support for using the KAP model to study medical staff of medical consortia. Nevertheless, it is recommended that further research be undertaken to investigate medical staff’s KAP regarding interdisciplinary or interdepartmental collaboration to promote operational efficiency and integrated health care in a medical consortium. Overall, this study shows that the attitudes toward and knowledge of the medical consortium significantly contribute to practices, satisfaction with the support work done by the leading hospital and agreement of improvement after joining the medical consortium. Thus, to improve medical staff’s KAP and satisfaction, publicity and educational programs in medical consortia are necessary, and the leading hospital should attach importance to the informatization construction and demand of different medical staff members.

### Electronic supplementary material

Below is the link to the electronic supplementary material.

Supplementary Material 1

## Data Availability

The data are not publicly available because the answers to the questions asked are personal assessments, which have been anonymized in this article. For data, please contact Wenqi Zeng (zwqi123@yeah.net).

## List of abbreviations

KAP: the knowledge, attitudes, and practices
WCH: West China Hospital of Sichuan University

## References

[1] Wang J, Jia W (2021). Resources allocation and utilization efficiency in China’s healthcare sector. China Finance Econ Rev.

[2] Anita W (2003). What is the medical alliance?. J Ky Med Assoc.

[3] Yip W, Hsiao W (2014). Harnessing the privatisation of China’s fragmented health-care delivery. Lancet.

[4] Verhulst J, Kramer D, Swann AC, Hale-Richlen B, Beahrs J (2013). The medical alliance: from placebo response to alliance effect. J Nerv Ment Dis.

[5] Office of the State Council. The general office of the state council issued the guiding opinions on promoting the construction and development of medical union. 2017. http://www.gov.cn/zhengce/content/2017-04/26/content_5189071.htm. Accessed 15 Nov 2022.

[6] Cai M, Liu E, Tao H, Qian Z, Fu QJ, Lin X, Wang M, Xu C, Ni Z (2018). Does a medical consortium influence health outcomes of hospitalized cancer patients? An integrated care model in Shanxi, China. Int J Integr Care.

[7] Yang F, Yang Y, Liao Z (2020). Evaluation and analysis for Chinese medical alliance’s governance structure modes based on preker-Harding model. Int J Integr Care.

[8] WHO (2015). WHO global strategy on people-centred and integrated health services: interim report.

[9] WHO (2016). Framework on integrated, people-centred health services: reported by the secretariat.

[10] Liang LL (2019). Impact of integrated healthcare: Taiwan’s family doctor plan. Health Policy Plan.

[11] Yue X, Mu K, Liu L (2020). Selection of policy instruments on integrated care in China: based on documents content analysis. Int J Environ Res Public Health.

[12] Ge J, Geng S, Jing L, Jiang H, Sun X (2019). Exploration and practice of the 1 + 10 + 1100000 model. Int J Health Plan Manag.

[13] Zou L, Chen X, Xu C, Xing L, Xie Y (2020). Design and preliminary experience of a tele-radiotherapy system for a medical alliance in China. Telemed J E Health.

[14] Shi G, Zhou B, Cai ZC, Wu T, Li XF, Xu W (2014). Referral by outreach specialist reduces hospitalisation costs of rural patients with digestive tract cancer: a report from medical consortium in China. Rural Remote Health.

[15] Song H, Zuo X, Cui C, Meng K (2019). The willingness of patients to make the first visit to primary care institutions and its influencing factors in Beijing medical alliances: a comparative study of Beijing’s medical resource-rich and scarce regions. BMC Health Serv Res.

[16] Schubert I, Siegel A, Graf E, Farin-Glattacker E, Ihle P, Köster I, Stelzer D, Mehl C, Schmitz J, Dröge P (2019). Study protocol for a quasi-experimental claims-based study evaluating 10-year results of the population-based integrated healthcare model ‘Gesundes Kinzigtal’ (healthy Kinzigtal): the INTEGRAL study. BMJ Open.

[17] Meaker R, Bhandal S, Roberts CM (2018). Information flow to enable integrated health care: integration or interoperability. Br J Gen Pract.

[18] Tham TY, Tran TL, Prueksaritanond S, Isidro JS, Setia S, Welluppillai V (2018). Integrated health care systems in Asia: an urgent necessity. Clin Interv Aging.

[19] Li Z, Zhang L, Pan Z, Zhang Y (2020). Research in integrated health care and publication trends from the perspective of global informatics. Gesundheitswesen.

[20] Gregg A, Tutek J, Leatherwood MD, Crawford W, Friend R, Crowther M, McKinney R (2019). Systematic review of community paramedicine and EMS mobile integrated health care interventions in the United States. Popul Health Manag.

[21] Ejeh FE, Saidu AS, Owoicho S, Maurice NA, Jauro S, Madukaji L, Okon KO (2020). Knowledge, attitude, and practice among healthcare workers towards COVID-19 outbreak in Nigeria. Heliyon.

[22] Abdelhafiz AS, Mohammed Z, Ibrahim ME, Ziady HH, Alorabi M, Ayyad M, Sultan EA (2020). Knowledge, perceptions, and attitude of egyptians towards the Novel Coronavirus Disease (COVID-19). J Community Health.

[23] Saqlain M, Munir MM, Rehman SU, Gulzar A, Naz S, Ahmed Z, Tahir AH, Mashhood M (2020). Knowledge, attitude, practice and perceived barriers among healthcare workers regarding COVID-19: a cross-sectional survey from Pakistan. J Hosp Infect.

[24] Alzghoul BI, Abdullah NA (2015). Pain management practices by nurses: an application of the knowledge, attitude and practices (KAP) model. Glob J Health Sci.

[25] AlMarzooqi LM, AlMajidi AA, AlHammadi AA, AlAli N, Khansaheb HH (2018). Knowledge, attitude, and practice of Influenza vaccine immunization among primary healthcare providers in Dubai health authority, 2016–2017. Hum Vaccin Immunother.

[26] Ahmed AM, Giang HTN, Ghozy S, Salem H, Algazar MO, Altibi A, Son HT, Nam Anh TH, Cuong TD, Tuan LQA (2019). Introduction of novel surgical techniques: a survey on knowledge, attitude, and practice of surgeons. Surg Innov.

[27] Abu-Amara TB, Al Rashed WA, Khandekar R, Qabha HM, Alosaimi FM, Alshuwayrikh AA, Almadi MK, Alfaris A (2019). Knowledge, attitude and practice among non-ophthalmic health care providers regarding eye management of diabetics in private sector of Riyadh, Saudi Arabia. BMC Health Serv Res.

[28] Feißel A, Peter R, Swart E, March S. Developing an extended model of the relation between work motivation and health as affected by the work ability as part of a corporate Age Management Approach. Int J Environ Res Public Health 2018, 15(4). 10.3390/ijerph15040779 PMC5923821 29673218

[29] Cai Y, Wen C, Tang L, Liu P, Xu Y, Hu S, Wei M, Cao J (2018). Exploration and consideration of the medical alliance modes. Iran J Public Health.

[30] Martinez RM (2019). Remote technical review of blood culture gram stains at a large integrated healthcare network. J Appl Lab Med.

[31] Khresheh R, Barclay L (2020). Knowledge, attitude and experience of episiotomy practice among obstetricians and midwives in Jordan. Women Birth.

[32] Huang M (2012). Key user knowledge, attitude and IT performance: the moderating effect of organizational culture. Procedia Eng.

[33] Phoosuwan N, Lundberg PC (2020). Knowledge, attitude and self-efficacy program intended to improve public health professionals’ ability to identify and manage perinatal depressive symptoms: a quasi-experimental study. BMC Public Health.

[34] Abdulsalam S, Ibrahim A, Saidu H, Muazu M, Aliyu UT, Umar HI, Gezawa ID, Owolabi LF (2018). Knowledge, attitude, and practice of diabetic retinopathy among physicians in Northwestern Nigeria. Niger J Clin Pract.

[35] Zacher H, Yang J. Organizational climate for successful aging. Front Psychol 2016, 7. 10.3389/fpsyg.2016.01007 PMC4930930 27458405

[36] Ng TWH, Feldman DC (2013). Age and innovation-related behavior: the joint moderating effects of supervisor undermining and proactive personality. J Organizational Behav.

[37] GBD 2016 Healthcare Access and Quality Collaborators (2018). Measuring performance on the healthcare access and quality index for 195 countries and territories and selected subnational locations: a systematic analysis from the global burden of Disease study 2016. Lancet.

[38] Li P, Li L, Zhang X. Regional Inequality of Firms’ Export Opportunity in China: Geographical Location and Economic Openness. In: Sustainability. vol. 12; 2020.

