# Supplementary material for: Perceived knowledge, attitudes and practices regarding the medical consortium among medical staff in Sichuan, China: a cross-sectional survey
Source: BMC Health Serv Res. 2023 Nov 29;23:1318. doi: 10.1186/s12913-023-10146-x (PMC10688012; doi:10.1186/s12913-023-10146-x)
Supplement: Supplementary file 1 — Supplementary Material 1 [file 12913_2023_10146_MOESM1_ESM.docx]

**Questionnaire about perceived knowledge, attitudes and practices of medical staff regarding the medical consortium**

This is an anonymous questionnaire survey. All research carried out was conducted with integrity and in line with generally accepted ethical principles, as well as approved by the Research Ethics Committee of West China Hospital of Sichuan University, Sichuan, China (internal registration number: HX 307/18).

I was informed about the study and provided consent allowing their data to be used for academic research.

1. Your gender:

□ Male □ Female

1. Your age (years): __________
2. Your length of service (years): __________
3. Your job type:

□ Doctors □ Nurses □ Medical technicians □ Administrators

1. Your education:

□ College □ Baccalaureate □ Master’s or higher

1. Your title:

□ Junior □ Intermediate □ Senior

Please answer the questions below using 1-5 (1 = strongly disagree/do not know, 5 = strongly agree/know, 2 and 4 represent moderate levels of disagreement/agreement respectively, 3 for a neutral or unsure response).

| **Items** | **Points** |
| --- | --- |
| **Perceived knowledge** |  |
| Knowing about the cooperation with other hospitals in the medical consortium (%) | YES / NO |
| Concept of the medical consortium | □ |
| Knowing what the leading hospital is doing to support the medical consortium | □ |
| A hierarchical medical system can be realized through the medical consortium | □ |
| **Attitudes** |  |
| Supporting the hospital to join a medical consortium | □ |
| Agreeing to the ward rounds and clinical teaching and training organized by the leading hospital | □ |
| Agreeing to the informatization construction in the medical consortium | □ |
| Agreeing to mutual recognition of inspection results in the medical consortium | □ |
| **Practices** |  |
| Frequency of participation in ward rounds and clinical teaching organized by the leading hospital | □ |
| Frequency of participation in activities organized by the leading hospital | □ |
| **Other questions** |  |
| Satisfaction about the support work done by the leading hospital for its members | □ |
| The hospital improved after joining the medical consortium | □ |

Note: It is worth noting that the original questionnaire was in Chinese, as this is the language respondents are most familiar with. We have invited experts proficient in both English and Chinese to review the revised translation in order to accurately present it to scholars fluent in English.

**Sample Institution Selection and Basic Situation**

This study selected the cross-regional medical consortia of Sichuan Province as the research scope, mainly based on the following considerations: (1) As a multi-ethnic province, Sichuan can be roughly divided into two regions: Western Sichuan, which is a vast, rural, mountainous, and sparsely populated high-altitude area; Eastern Sichuan, which has a dense population, developed highway networks and economy, and is a plain area. This geographical, demographic and economic distribution also widely reflects the situation across the whole country. Sichuan Province also has the situation of obvious imbalanced distribution of medical resources. Taking Sichuan Province as the research area nationwide has typical significance; (2) With the comprehensive advancement of national policies, the number of medical consortia has gradually increased, but there is a large gap between eastern and western regions. Taking the cross-regional medical consortia in Sichuan Province as the research scope can provide reference and inspiration for the construction of medical consortia in western regions.

We take the lead medical consortia model of West China Hospital of Sichuan University as the research object. Considering the time lag effect of implementation effects (i.e. there is a certain lag time difference between policy implementation and significant policy effects), we selected 3 alliance hospitals that were early practitioners of cross-regional medical consortia as sample hospitals, which are Hospital G, Hospital Z, and Hospital J in Chengdu. The basic information of West China Hospital, sample hospitals and their locations are shown in Table 1.

**Table 1 Basic information of West China Hospital, sample hospitals and their locations**

| Project | West China Hospital | Hospital G | Hospital Z | Hospital J |
| --- | --- | --- | --- | --- |
| Hospital level | Tertiary Grade A | Tertiary Grade A | Tertiary Grade A | Tertiary Grade B |
| Time of consortium implementation | — | 2015 | 2016 | 2016 |
| Distance from West China Hospital (km) | — | 288.5 | 101.1 | 62.1 |
| Per capita GRP of location (yuan) | 94,782 | 38,520 | 42,112 | 59,939 |
| Number of health institutions in location | 10,755 | 3,446 | 3,438 | 522 |
| Number of beds per 1,000 population in location | 8.77 | 6.10 | 7.57 | 7.41 |
| Number of health technicians per 1,000 population in location | 10.35 | 4.87 | 5.74 | 5.07 |

Data source: Sichuan Statistical Yearbook 2019; Statistical Bulletin of Chengdu's National Economic and Social Development in 2018; 2018 Statistical Bulletins of National Economic and Social Development of City G, City Z, and County J; Real-time query on Baidu Maps

Note: Implementation time refers to the time when the alliance hospital joined the lead medical consortia of West China Hospital and was officially named as Hospital XX of West China Hospital, Sichuan University; Per capita GRP = Per capita gross regional product

The three sample hospitals are leading local hospitals in their respective regions. After being directly supported and aided by West China Hospital, they have further driven medical institutions at all levels in the region to form a graded collaborative medical service network of "1+1+X", and the regional medical consortia radiation networks of the three hospitals are as follows:

Hospital G has led 33 medical institutions within its jurisdiction to participate in the construction of the medical consortia, covering all counties and districts of the city. It includes 3 tertiary hospitals, 11 secondary hospitals, 15 central health centers, 1 other grade hospital, 3 private hospitals, and has established 1 telemedicine collaboration network, with 11 medical institutions joining. It has established 2 regional medical centers, including a GCP (Good Clinical Practice) center and a stroke center.

Hospital Z has established cooperative relationships with 25 medical institutions within its jurisdiction (counterpart support, technical collaboration and two-way referral), including 2 county hospitals, 1 district hospital, 1 community health service center, 17 township health centers, and 4 other category hospitals (2 private hospitals, 1 social welfare institution, 1 employee hospital). A remote consultation center has been established in the region.

Hospital J has led 11 township health centers and 1 community health service center in the region to participate in the construction of the medical consortia. It has also established 1 chest pain center and 1 stroke center. In addition, it is building imaging centers, electrocardiogram centers, inspection centers, and MDT (Multiple Disciplinary Team) centers.
